# PERSPECTIVES ON TECHNOLOGY ADOPTION AMONG SPANISH-SPEAKING HISPANIC ADULTS AGE 60+

**DOI:** 10.1093/geroni/igad104.2866

**Published:** 2023-12-21

**Authors:** Sheila Salinas Navarro, Sindy Lomeli, Lilly Estenson, Kathleen Wilber

**Affiliations:** University of Southern California, Los Angeles, California, United States; University of Southern California, Los Angeles, California, United States; University of Southern California, Los Angeles, California, United States; University of Southern California, Los Angeles, California, United States

## Abstract

By 2060, Hispanics aged 65+ are projected to be 21% (19.9 million) of the total US age 65+ population. Despite the benefits of technology use, such as facilitating social engagement and access to health services, few studies have explored attitudes of older Spanish-Speaking Hispanic adults toward technology adoption. In this secondary analysis of a larger diverse study of attitudes toward technology, we reviewed audio recordings and transcripts of Spanish-language interviews and focus groups with adults ages 63-89. Participants (n=21) self-identified as Hispanic, 67% had less than a high school education, and almost half (n=10) lived alone. Connecting with family members abroad was the primary motivator and a “necessity” for using technology; excess use of a device was negative. As one participant stated: “Technology brings you closer to those far away and pushes you away from those near you.” Those without relatives living with or near them reported greater difficulty using technology and identified living alone as a barrier to adoption. Finally, although the sample had low levels of English proficiency and low educational attainment, characteristics associated with lower use and knowledge of technology, findings challenge stereotypes that these older adults lack the capacity or interest to adopt the technology. Rather, the study identified multiple social factors that may hinder adoption. Understanding these cultural and linguistic barriers is important to promote digital inclusion; overcoming the digital divide will involve more than providing free tablets or classes. It will also require addressing barriers with adequate, culturally competent understanding, training and support.

